# Data Management in Biobanking: Strategies, Challenges, and Future Directions

**DOI:** 10.3390/biotech13030034

**Published:** 2024-09-02

**Authors:** Ramez Alkhatib, Karoline I. Gaede

**Affiliations:** 1 Biomaterial Bank Nord, Research Center Borstel Leibniz Lung Center, Parkallee 35, 23845 Borstel, Germany; kgaede@fz-borstel.de; 2 German Centre for Lung Research (DZL), Airway Research Centre North (ARCN), 22927 Großhansdorf, Germany; 3 PopGen 2.0 Biobanking Network (P2N), University Hospital Schleswig-Holstein, Campus Kiel, Kiel University, 24105 Kiel, Germany

**Keywords:** biobanking, data management, ethics in biobanking, data quality, data integration, data standardization, data sharing, data privacy, data governance

## Abstract

Biobanking plays a pivotal role in biomedical research by providing standardized processing, precise storing, and management of biological sample collections along with the associated data. Effective data management is a prerequisite to ensure the integrity, quality, and accessibility of these resources. This review provides a current landscape of data management in biobanking, discussing key challenges, existing strategies, and potential future directions. We explore multiple aspects of data management, including data collection, storage, curation, sharing, and ethical considerations. By examining the evolving technologies and methodologies in biobanking, we aim to provide insights into addressing the complexities and maximizing the utility of biobank data for research and clinical applications.

## 1. Introduction

Biobanks are indispensable pillars in biomedical research, serving as repositories for a vast range of biological specimens and the associated data [1]. These repositories hold immense potential to revolutionize our understanding of health and disease, offering researchers invaluable resources for studying genetic, molecular, and environmental factors that influence human health [2,3]. The foundation of biobanking lies in the collection, storage, and management of diverse biospecimens, ranging from tissue samples and blood specimens to genetic data and clinical/phenotyping information [4].

In recent years, the significance of biobanks has grown exponentially, driven by advancements in technology, the increasing complexity of research questions, and the emergence of precision medicine initiatives. Biobanks not only provide researchers with access to a rich tapestry of biological samples but also offer a treasure trove of associated data, including demographic information, medical histories, and genetic profiles [5]. These datasets hold the potential to unravel disease mechanisms, identify biomarkers for early diagnosis and prognosis, and personalize treatment strategies for improved patient outcomes [6].

However, the importance of biobanks is not solely determined by the quantity of specimens stored but also heavily relies on the quality, integrity, and accessibility of the related data. Implementing effective data management protocols is crucial to maintaining the value of biobank resources for the scientific community [7,8]. Challenges such as data heterogeneity, quality assurance, privacy concerns, and regulatory compliance underscore the complexities inherent in managing biobank data [9].

This review offers a multifaceted landscape of data management in biobanking, examining the challenges faced, the strategies employed, and the future directions envisioned. By elucidating the critical role of data management in increasing the utilization of biobank resources, we aim to shed light on the importance of robust data management practices in advancing biomedical research and ultimately improving human health.

## 2. Biospecimens

Biological specimens are the foundation of biobanks, which are crucial facilities that store a wide range of biological samples that are received from donors. These specimens are essential for research projects, ranging from disease understanding to the development of novel treatments. Biosamples are indispensable tools in research on genetic variations, biomarkers, pathomechanisms, and therapy, enhancing healthcare outcomes. This section thoroughly examines the central role of biological specimens within biobanks.

### 2.1. Importance of Biospecimens

Biospecimens are crucial resources in medical research, playing a central role in increasing the knowledge on human health and disease. The understanding of pathomechanisms is significantly enhanced by unique and well-characterized biospecimens from deeply phenotyped individuals with defined health conditions. These specimens are essential for investigating the molecular mechanisms underlying diseases, identifying biomarkers for early disease detection, and elucidating pathways for potential therapeutic interventions. For instance, the analysis of cancerous tissue samples can unveil genetic mutations fueling tumor growth, facilitating the development of precisely targeted treatments [10]. Biospecimens also drive personalized medicine, as personalized and precision medicine rely heavily on biological specimens. By analyzing individual genetic profiles, biomarker expression, and other molecular attributes, healthcare providers can tailor treatments to match each patient’s unique characteristics. Biospecimens enable the identification of predictive markers for drug responses and disease progression, leading to more precise and personalized healthcare interventions [11].

### 2.2. Types of Biospecimens

**Figure 1:**
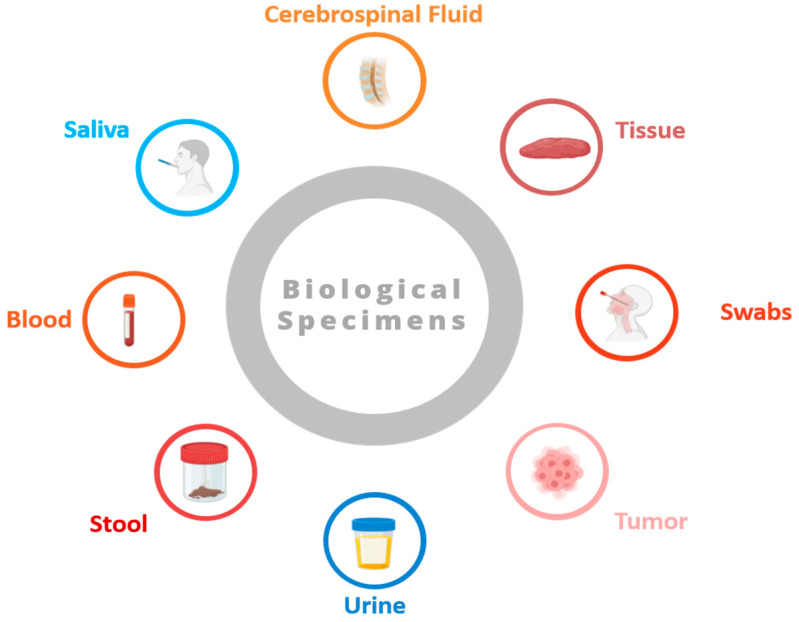
Some common types of biospecimens (figure contains BioRender icons).

Human biospecimens offer medical research unique insights into broad aspects of health, disease development, and treatment [3]. Listed here are some common types of biospecimens (see Figure 1):

- Blood samples: Blood plays a crucial role in the body, transporting oxygen, nutrients, hormones, and waste products. Obtained through procedures like venipuncture or finger pricking, blood samples are rich in information, containing details like blood cell counts, biochemical markers, hormones, and genetic material (DNA and RNA). They are utilized across various medical fields for diagnostics, disease tracking, and research endeavors.
- Tissue biopsies: Tissue biopsies involve extracting small tissue samples from organs or lesions for microscopic examination. These samples provide vital diagnostic insights, enabling pathologists to identify cellular irregularities, tissue structures, and molecular markers associated with conditions such as cancer, infections, and autoimmune disorders. Techniques like needle biopsies, surgical excision, and endoscopic procedures are employed to obtain tissue biopsies.
- Saliva and oral swabs: Saliva and oral swabs contain a mix of cells, enzymes, proteins, and microorganisms that are present in the oral cavity. These specimens are collected non-invasively and are employed to study oral health, detect oral pathogens, and analyze the oral microbiome. Saliva samples also offer insights into systemic conditions like diabetes, cardiovascular disease, and autoimmune disorders. Oral swabs find utility in genetic testing and forensic analysis.
- Urine samples: Urine, a waste product produced by the kidneys, holds metabolic byproducts, electrolytes, hormones, and other substances filtered from the blood. Routinely collected for urinalysis, urine samples help evaluate the kidney function, hydration status, and presence of abnormalities such as urinary tract infections, kidney stones, and proteinuria. They are also utilized in drug screening, pregnancy testing, and research studies.
- Stool samples: Stool, or feces, is the waste product expelled from the gastrointestinal tract. Stool samples contain undigested food, water, bacteria, viruses, and other substances. Collected for diagnostic purposes, they help detect gastrointestinal infections, evaluate digestive function, and screen for colorectal cancer. Stool samples are also used to explore the gut microbiome, digestive disorders, and inflammatory bowel diseases.

## 3. Data Types in Biobanking

**Figure 2:**
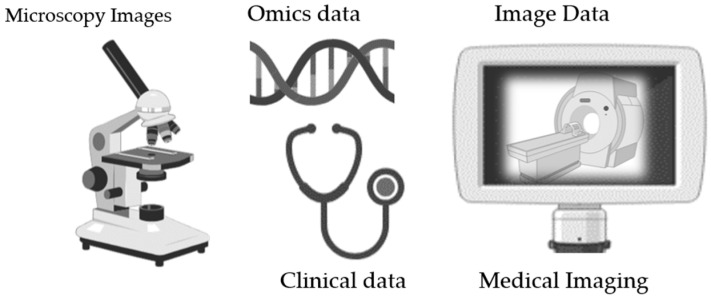
Data types in biobanking (figure contains BioRender icons).

Biobanking encompasses a diverse range of biological specimens obtained from individuals, which are accompanied by comprehensive clinical, demographic, environmental, and molecular data. Understanding the broad spectrum of data types within biobanks is crucial for maximizing their utility in both biomedical research and clinical practice [12,13]. This section delves into the multifaceted nature of the data stored in biobanks and its critical role in advancing scientific knowledge and healthcare outcomes. In addition to the conventional biological specimens and linked clinical records, biobanks are increasingly incorporating image data as an invaluable asset for biomedical exploration and clinical diagnostics [14,15]. Ranging from histopathological slides to various medical imaging modalities such as MRI, CT scans, and microscopic imaging [16], visual datasets provide a distinctive viewpoint on biological structures, functions, and disease presentations [17,18] (see Figure 2). 

### 3.1. Clinical Data

Clinical data, encompassing the clinical phenotype, furnishes vital insights into patients’ medical backgrounds, diagnoses, treatments, and prognoses and is indispensable for biobanks. This encompasses demographic particulars such as age, gender, and ethnicity, as well as environmental and lifestyle factors. Additionally, clinical metrics such as disease status, pathology findings, imaging results, and therapeutic regimens are crucial. By aligning biological specimens with detailed clinical annotations, biobanks empower researchers to delve into disease origins, progression, and therapeutic responses with heightened precision and granularity.

### 3.2. Image Data

Here are some common types of image data that can be stored in biobanking:

- Histopathological images: Histopathological images capture tissue samples stained with diverse dyes to visualize cellular structures and arrangements. These images are pivotal in disease diagnosis, tumor evaluation, and prognostic assessment. Biobanks maintain archives of histopathological slides alongside detailed clinical annotations, empowering researchers to correlate histological characteristics with molecular profiles and clinical outcomes.
- Medical imaging: Medical imaging encompasses a plethora of techniques including MRI, CT scans, PET scans, ultrasound, X-rays, and thermal imaging, facilitating the non-invasive visualization of anatomical structures, physiological activities, and pathological changes in living organisms. Biobanks curate repositories of medical imaging data obtained from routine clinical procedures, research studies, and clinical trials, enabling retrospective analyses and longitudinal investigations across diverse patient cohorts [19,20].
- Microscopy images: Microscopy images capture intricate cellular and subcellular structures with remarkable resolution, providing insights into cellular morphologies, spatial organizations, and dynamic processes. Biobanks preserve microscopy images that are acquired through various techniques such as light microscopy, electron microscopy, and confocal microscopy, supporting research endeavors in fields such as cell biology, neuroscience, and developmental biology. These images facilitate quantitative analyses of cellular phenotypes, protein distributions, and cellular interactions in both healthy and diseased states.

### 3.3. Omics Data

There are various types of data that can be considered as omics data:

- Genomic data, encapsulating DNA sequences, variations, and structural nuances, constitute an indispensable facet of biobanking. Driven by advances in high-throughput sequencing technologies, biobanks house diverse genomic datasets spanning entire genomes, exomes, and genotyping arrays. These datasets facilitate genome-wide association studies (GWASs), variant exploration, and pharmacogenomic investigations, with the integration of genomic data and clinical insights holding promise for deciphering genotype–phenotype relationships and guiding tailored treatment approaches.
- Transcriptomic data: Transcriptomic data capture the expression profiles of genes under various biological conditions, unraveling intricate cellular processes and regulatory networks. Biobanks curate transcriptomic datasets derived from methodologies like microarrays and RNA sequencing (RNA-seq), enabling researchers to probe gene expression patterns linked to disease states, tissue phenotypes, and therapeutic responses. Transcriptomic analyses of biobanked specimens drive biomarker discovery, target identification, and mechanistic inquiries across diverse domains spanning oncology to neurology.
- Proteomic data: Proteomic data entail the identification and quantification of proteins within biological samples, offering a snapshot of their cellular functions and signaling pathways. Biobanks store proteomic datasets derived from mass spectrometry-based techniques, immunoassays, and protein arrays, facilitating the characterization of protein expression, modifications, and interactions. The integration of proteomic insights with other omics layers enriches our understanding of disease mechanisms, biomarker profiles, and treatment responses, thereby paving the way for precise therapeutic interventions.
- Metabolomic data: Metabolomic data capture the repertoire of small-molecule metabolites within biological samples, serving as mirrors of cellular metabolism and biochemical pathways. Biobanks archive metabolomic profiles obtained using methodologies like nuclear magnetic resonance (NMR) spectroscopy and liquid chromatography–mass spectrometry (LC-MS), enabling investigations into metabolic dysregulations across diseases such as cancer, metabolic disorders, and neurodegenerative conditions. The integration of metabolomic signatures with other omics datasets furnishes holistic insights into disease phenotypes and metabolic imbalances underpinning health and disease.

## 4. Challenges in Data Management

Data management encompasses the various challenges that biobank institutions encounter in handling, storing, and utilizing data effectively [21,22,23,24,25]. Here are some common challenges:

### 4.1. Data Heterogeneity

Data heterogeneity poses a significant challenge in data management within biobanking, primarily due to the diverse nature of biological samples and the associated data collected and stored in these repositories. Here, we provide an overview of the complexities of data heterogeneity and its implications for effective data management in biobanking.

- Diverse data types: Biobanks collect a wide range of biological samples, including tissues, blood, urine, and cells, each with its unique characteristics and properties. Furthermore, the associated data encompass a wide range of data types, including genomic data, clinical records, imaging data, and information on environmental exposure. Managing such diverse datasets requires robust systems capable of handling multiple data formats, structures, and standards [26].
- Varying data standards: Different biobanks may adhere to varying data standards, terminology, and annotation protocols, leading to inconsistencies in data representation and interoperability challenges. Harmonizing data across multiple biobanks and research studies becomes inherently challenging due to the lack of standardized practices for data collection, annotation, and storage.
- Data annotation and metadata: Effective data management relies on accurate metadata annotation to provide context and interpretability to the stored data. However, the heterogeneity of data sources often results in incomplete or inconsistent metadata, making it challenging to interpret and analyze the data accurately. Standardizing metadata annotation practices is essential for ensuring data integrity and facilitating data integration across different biobanks and research projects.
- Integration and interoperability: Integrating heterogeneous datasets from multiple sources is crucial for conducting comprehensive analyses and deriving meaningful insights. However, data heterogeneity complicates the integration process, requiring sophisticated data integration methods and tools to reconcile the differences in data formats, semantics, and ontologies. Achieving interoperability across disparate datasets is essential for promoting data sharing and collaboration in the scientific community.
- Data quality and reliability: Heterogeneous data sources may vary in quality, completeness, and reliability, posing challenges for ensuring data accuracy and consistency. Quality control measures must be implemented throughout the data lifecycle to identify and rectify errors, outliers, and inconsistencies. Data validation, cleaning, and normalization techniques are essential for maintaining data quality and reliability, particularly in large-scale biobanking initiatives.
- Ethical and legal considerations: Data heterogeneity also extends to ethical and legal considerations surrounding data privacy, consent, and ownership. Harmonizing ethical standards and regulatory requirements across different jurisdictions is essential to ensure adherence to data protection regulations like GDPR and HIPAA.

### 4.2. Data Quality Assurance

Ensuring data quality is a main issue in biobanking, where accuracy, completeness, and reliability are essential for maintaining the integrity and utility of stored biological specimens and their associated data. Here, we delve into the challenges posed by data quality assurance in biobanking data management:

- Sample integrity and traceability: Biobanks must maintain the integrity and traceability of biological samples throughout their lifecycle, from collection to storage and distribution. Ensuring proper sample handling, storage conditions, and chain-of-custody protocols is crucial for preventing sample degradation, contamination, or mislabeling, which could compromise data quality and research outcomes.
- Data accuracy and consistency: The data collected and curated in biobanks must be accurate, consistent, and reliable to support meaningful research conclusions. However, data entry errors, inconsistencies in data annotation, and discrepancies between different data sources can introduce inaccuracies and biases into the dataset. Implementing data validation checks, standardizing data entry procedures, and conducting regular data audits are imperative for upholding data accuracy and consistency.
- Missing data and incomplete records: Incomplete or missing data entries are common challenges in biobanking, where data may be unavailable or incomplete due to various reasons such as sample collection limitations, participant non-compliance, or data entry errors. Addressing missing data requires robust data imputation techniques and strategies for data completeness assessment. Additionally, establishing protocols for documenting missing data and mitigating its impact on research outcomes is essential for maintaining data quality.
- Data reconciliation and harmonization: Biobanks often aggregate data from multiple sources, including clinical records, laboratory measurements, and genetic analyses. Reconciling and harmonizing heterogeneous data sources to ensure consistency and interoperability pose significant challenges. Establishing standardized data formats, vocabularies, and ontologies, along with data normalization and transformation techniques, is essential for integrating diverse datasets while maintaining data quality.
- Quality control processes: Implementing rigorous quality control processes is crucial for identifying and rectifying data errors, outliers, and inconsistencies. Quality control measures might encompass data validation checks, data cleaning procedures, and outlier detection algorithms, all aimed at ensuring the integrity and reliability of the data. Regular quality assessments and audits help monitor data quality over time and ensure adherence to established quality standards.
- Long-term data preservation: Preserving data integrity and accessibility over the long term presents a considerable challenge for biobanks, particularly as technology and data formats evolve over time. Establishing robust data stewardship and preservation strategies, including data backup, version control, and migration plans, is essential for safeguarding data integrity and ensuring their longevity for future research endeavors.
- Ethical and regulatory compliance: Data quality assurance in biobanking needs to adhere to ethical principles and regulatory requirements governing participant privacy, consent, and data protection. Implementing data governance frameworks, privacy safeguards, and security measures is essential for compliance with legal and ethical guidelines such as GDPR [27] and HIPAA while maintaining data quality and integrity.

### 4.3. Privacy and Security

Privacy and security represent paramount considerations in biobanking data management, owing to the sensitive nature of the data involved and the potential risks linked to unauthorized access or breaches. Here, we explore the challenges posed by privacy and security in biobanking data management:

- Participant confidentiality: Biobanks hold considerable amounts of data containing sensitive information about participants, including personal identifiers, medical histories, and genetic profiles. Ensuring participant confidentiality and protecting privacy rights are fundamental ethical principles in biobanking. However, the amount and diversity of the data increase the risk of unintended disclosures or privacy breaches, necessitating robust privacy safeguards and access controls.
- Encryption and access management: Deploying robust encryption protocols and access management systems is crucial for safeguarding biobank data against unauthorized access or breaches. Encryption methods like data-at-rest and data-in-transit encryption serve to secure data both during storage on servers and while they are being transmitted. Access management strategies, such as role-based access control (RBAC) and multi-factor authentication (MFA), limit access solely to authorized individuals, thereby reducing the potential for insider threats.
- Data anonymization and de-identification: Anonymizing or de-identifying data represents a prevalent approach in biobanking, aiming to safeguard participant privacy while retaining data usefulness for research endeavors. However, achieving true anonymity or irreversibility poses challenges, as re-identification risks remain, especially with the proliferation of data linkage and re-identification techniques. Balancing data anonymization with data utility requires the careful consideration of anonymization methods and privacy-preserving techniques.
- Data sharing and consent management: Facilitating data sharing while respecting participant consent preferences is a complex undertaking in biobanking. Ensuring that participants have meaningful control over their data and understanding how their data will be used is essential for fostering trust and transparency. Implementing robust consent management systems, including dynamic consent models and granular consent options, enables participants to specify their preferences regarding data sharing and use.
- Regulatory compliance: Biobanking data management must comply with a myriad of legal and regulatory requirements governing data privacy and security, including General Data Protection Regulation (GDPR) [28], Health Insurance Portability and Accountability Act (HIPAA) [29], and other data protection laws. Adhering to regulatory standards requires implementing comprehensive data governance frameworks, conducting privacy impact assessments, and maintaining documentation of data processing activities. Failure to comply can lead to significant penalties and harm to the reputation of biobanks.
- Data breach preparedness and response: Despite best efforts to prevent breaches, biobanks need to be ready to react promptly and efficiently in case of a data breach. Establishing incident response plans, including procedures for breach notification, forensic investigation, and communication with affected parties, is crucial for mitigating the impact of breaches on participant privacy and trust.
- Data lifecycle management: Ensuring the effective management of data from its collection to disposal necessitates the implementation of robust data management practices that prioritize privacy and security. Implementing data minimization strategies, secure data disposal procedures, and audit trails for data access and usage enhances accountability and mitigates the risk of unauthorized data exposure

### 4.4. Data Governance and Regulatory Compliance

Data governance and regulatory compliance represent significant challenges in biobanking data management, as they involve navigating a complex landscape of legal and ethical requirements while ensuring the responsible stewardship of data. Here, we delve into the multifaceted challenges posed by data governance and regulatory compliance in biobanking:

- Legal and ethical frameworks: Biobanks operate within a framework of legal and ethical guidelines that govern the collection, storage, and use of biological samples and their associated data. Adherence to regulations like the GDPR and HIPAA as well as the ethical principles outlined in documents like the Declaration of Helsinki are prerequisites for the protection of participant rights and ensuring research integrity.
- Informed consent and participant privacy: Obtaining informed consent from participants is a cornerstone of ethical biobanking practices, guaranteeing that individuals comprehend the objectives of data collection, the intended utilization of their data, and any potential risks inherent in the process [4]. However, obtaining meaningful consent can be challenging, especially in longitudinal studies or when data may be used for future, unforeseen research purposes. Balancing participant autonomy with the need for scientific advancement requires clear communication and consent management strategies.
- Data ownership and intellectual property: Elucidating rights to data ownership and addressing intellectual property concerns is essential for resolving legal and ethical issues surrounding data usage, access, and commercialization. Biobanks often navigate complex relationships between participants, researchers, institutions, and commercial entities, necessitating clear policies and agreements regarding data ownership, sharing, and commercialization rights.
- Data access and sharing policies: Establishing transparent data access and sharing policies is essential for promoting research collaboration, maximizing data utility, and ensuring equitable access to biobank resources. However, balancing openness with privacy concerns and intellectual property rights poses challenges, particularly when sharing data across international borders or with commercial partners. Implementing access control mechanisms and data use agreements helps regulate data access while protecting participant privacy and confidentiality.
- Data security and confidentiality: Protecting the security and confidentiality of biobank data is a legal and ethical imperative, requiring robust data security measures and safeguards against unauthorized access or breaches. Adhering to data protection regulations like GDPR and HIPAA entails implementing encryption, access controls, and data anonymization techniques to mitigate privacy risks and safeguard participant confidentiality.
- Audit and compliance monitoring: Monitoring compliance with data governance policies and regulatory requirements requires robust audit mechanisms and oversight processes. Conducting regular audits of data management practices, documentation, and security controls helps identify potential compliance gaps and mitigate risks of non-compliance. Establishing clear lines of accountability and oversight responsibilities is essential for ensuring adherence to regulatory standards.
- Data retention and disposal: Developing policies for data retention and disposal is essential for effectively managing the data lifecycle and minimizing privacy risks. Determining appropriate retention periods, archival strategies, and secure data disposal procedures requires the consideration of legal requirements, research needs, and participant consent preferences. Implementing data minimization principles and regular data purging practices reduces the risk of unauthorized data exposure and facilitates compliance with data protection laws.

## 5. Strategies for Effective Data Management

Efficient data management is vital for biobank institutions to harness the full potential of their data as a strategic asset. Here are several approaches to achieve this:

### 5.1. Standardization and Metadata Annotation

Standardization and metadata annotation are pivotal strategies for effective data management in biobanking, aimed to promote interoperability, facilitate data integration, and enhance data usability. Here, we outline the importance of standardization and metadata annotation and their roles in overcoming data management challenges:

- Data standardization: Standardizing data formats, vocabularies, and ontologies is essential for ensuring consistency and interoperability across the diverse datasets collected and stored in biobanks [30]. With the adoption of common data standards and terminologies, biobanks facilitate data sharing, integration, and reusability across multiple research studies and platforms [31,32]. Standardization efforts encompass various aspects of data management, including sample metadata, clinical annotations, genomic data formats, and laboratory measurements [33,34].
- Harmonization of data: Harmonizing heterogeneous datasets from different sources involves reconciling the differences in data formats, semantics, and structures to enable seamless data integration and analysis. Harmonization efforts aim to ensure that the data collected across multiple biobanks or research studies are compatible and comparable, thereby maximizing the utility of aggregated datasets for research purposes. Establishing harmonization guidelines, mapping protocols, and data transformation procedures helps address discrepancies and inconsistencies in data representation [35].
- Metadata annotation: Metadata annotation provides essential context and descriptive information about biological samples and their associated data, enhancing data interpretability and usability. Metadata encompass a wide range of attributes, including sample characteristics, experimental protocols, data provenance, and quality metrics. Annotating data with standardized metadata terms and controlled vocabularies enables researchers to search, filter, and analyze data effectively, facilitating data discovery and interpretation [36,37].
- Data integration platforms: Leveraging data integration platforms and bioinformatics tools streamlines the process of harmonizing and annotating heterogeneous datasets in biobanking. These platforms provide capabilities for data mapping, transformation, and enrichment, enabling researchers to aggregate, query, and analyze diverse datasets from multiple sources. By providing a unified interface for data access and analysis, data integration platforms promote collaboration, accelerate research discoveries, and maximize the value of biobank resources [38].
- Ontology development and adoption: Ontologies play a crucial role in standardizing and formalizing knowledge representation in biobanking, enabling semantic interoperability and data integration [39]. Ontologies provide structured vocabularies and hierarchical relationships for annotating biological concepts, phenotypic traits, and experimental variables [40]. Adopting community-developed ontologies, such as the Human Phenotype Ontology (HPO) or the Experimental Factor Ontology (EFO), facilitates data annotation and enhances data interoperability across different biobanks and research domains.
- Metadata quality assurance: Ensuring the quality and completeness of metadata annotations is essential for maintaining data integrity and facilitating accurate data interpretation. Metadata quality assurance measures include validation checks, consistency audits, and adherence to metadata standards and best practices. Establishing metadata curation guidelines, metadata validation rules, and quality control procedures helps mitigate errors and inconsistencies in metadata annotations, enhancing the reliability and usability of biobank data.
- Community engagement and collaboration: Collaborative efforts within the scientific community are crucial for driving standardization and metadata annotation initiatives in biobanking. Engaging stakeholders, including researchers, data scientists, informaticians, and domain experts, fosters consensus building, promotes knowledge sharing, and accelerates the adoption of standardized data management practices. Community-driven initiatives, such as data standards consortia, working groups, and data harmonization projects, play a vital role in advancing data standardization and metadata annotation efforts across the biobanking community.

### 5.2. Data Quality Control

Ensuring the accuracy, completeness, and reliability of data in biobanking is crucial for research integrity and maximizing the utility of stored biological specimens and their associated data. Here, we delve into the significance of data quality management and methods for its implementation:

- Data validation: Data validation verifies the data’s accuracy, consistency, and integrity through systematic checks and predefined criteria. These checks, conducted at data entry or import, identify errors, anomalies, and inconsistencies such as missing values or outliers, ensuring only high-quality data are inputted into the system.
- Quality assurance protocols: Developing quality assurance protocols and standard operating procedures (SOPs) are essential for the maintenance of consistent data quality standards across biobank operations. SOPs define procedures for data collection, storage, curation, and documentation, ensuring adherence to best practices and regulatory requirements. Regular training and audits help enforce compliance with quality assurance protocols and identify areas for improvement.
- Data cleaning and transformation: Data cleaning addresses errors, inconsistencies, and outliers in the dataset to enhance data quality and reliability. Cleaning procedures may include data deduplication, outlier detection, imputation of missing values, and normalization of data formats. Data transformation techniques, such as standardization or log transformation, help prepare data for analysis and mitigate biases introduced by data heterogeneity.
- Standardized data entry and documentation: Standardizing data entry procedures and documentation formats promotes consistency and accuracy in data collection and annotation. Providing clear guidelines, data dictionaries, and templates for data entry facilitates uniform data capture and ensures that relevant metadata are documented consistently [41,42]. Validating data against predefined data standards and vocabularies further enhances data quality and interoperability.
- Automated quality control checks: Implementing automated quality control checks and algorithms helps streamline data validation and cleaning processes, reducing manual effort and human errors. Automated checks may include range validation, format validation, and logical consistency checks to flag potential data anomalies in real time. Integrating automated quality control checks into data management workflows improves efficiency and ensures timely detection and resolution of data issues.
- Continuous monitoring and improvement: Data quality control is an ongoing process that requires continuous monitoring and enhancement to maintain data integrity over time. Monitoring data quality metrics like data completeness, accuracy rates, and error frequencies allows biobanks to evaluate the effectiveness of quality control measures and identify areas for optimization. Establishing feedback mechanisms and quality improvement initiatives fosters a culture of continuous quality improvement and enhances the reliability of biobank data.
- External quality assessment programs: Participating in external quality assessment programs and proficiency testing schemes provides independent validation of data quality and performance against established benchmarks and standards. External assessments help benchmark biobank performance, identify areas for improvement, and demonstrate compliance with regulatory requirements and accreditation standards. Engaging in collaborative quality assurance initiatives strengthens the credibility and trustworthiness of biobank data within the scientific community.

### 5.3. Secure Data Infrastructure

Secure data infrastructure is a central strategy for effective data management in biobanking and is essential for protecting the confidentiality, integrity, and availability of sensitive biological specimens and their associated data [25,43]. Here, we delve into the importance of secure data infrastructure and key strategies for its implementation:

- Data encryption: Deploying strong encryption methods for data, both at rest and in transit, serves to protect biobank data from unauthorized access or interception. Encryption standards such as the Advanced Encryption Standard (AES) for data storage and Transport Layer Security (TLS) for data transmission ensure that data remain encrypted and indecipherable to unauthorized parties, thus mitigating the risk of data breaches or interception during transmission.
- Access control and authentication: Establishing policies for access control and authentication mechanisms is essential in governing access to biobank data, ensuring that only authorized personnel can access sensitive information. Role-based access control (RBAC), multi-factor authentication (MFA), and stringent password policies serve to limit access to data based on user roles, privileges, and authentication credentials, thereby reducing the risk of unauthorized data access or insider threats.
- Data segregation and isolation: The segregation and isolation of sensitive data within secure environments, such as secure servers or dedicated data centers, help to thwart unauthorized access or tampering with biobank data. The implementation of network segmentation, firewalls, and intrusion detection systems (IDSs) effectively separates sensitive data from less secure networks, minimizing the impact of security breaches or cyberattacks on biobank operations.
- Secure data storage and backup: Employing secure data storage solutions, such as encrypted databases or cloud storage with integrated encryption and access controls, serves to safeguard biobank data from loss, theft, or corruption. Regular data backups and comprehensive disaster recovery plans ensure data resilience and enable swift data recovery in the event of hardware failures, natural disasters, or ransomware attacks, thereby minimizing downtime and potential data loss.
- Data masking and anonymization: Applying data masking or anonymization techniques to sensitive data helps protect participant privacy and confidentiality while preserving data utility for research purposes. Masking personally identifiable information (PII) or de-identifying data before sharing or analysis reduces the risk of re-identification and unauthorized disclosure of sensitive information, ensuring compliance with privacy regulations and ethical guidelines.
- Auditing and monitoring: Integrating robust auditing and monitoring mechanisms empowers biobanks to monitor data access, usage, and modifications, facilitating accountability and compliance with data governance policies. Audit trails, logging mechanisms, and real-time monitoring tools offer visibility into data activities and aid in detecting anomalous behavior or security incidents, enabling prompt response and remediation.
- Security awareness and training: Promoting security awareness and providing training to personnel on security best practices, data handling procedures, and incident response protocols is crucial for fostering a culture of security within the biobank. Educating staff about potential security risks, phishing attacks, and social engineering tactics helps mitigate human errors and strengthens defenses against cybersecurity threats, enhancing overall data security posture.
- Regulatory compliance and certifications: Ensuring compliance with regulatory requirements, such as GDPR, HIPAA, and ISO/IEC 27001 [9], demonstrates commitment to data security and privacy best practices. Obtaining certifications and undergoing independent audits validate a biobank’s adherence to industry standards and regulatory guidelines, instilling confidence in data security practices among stakeholders, researchers, and participants.

### 5.4. Data Sharing and Collaboration

Data sharing and collaboration are essential strategies for effective data management in biobanking, enabling researchers to maximize the utility of biological specimens and their associated data for advancing scientific discoveries and improving healthcare outcomes [23]. Here, we outline the importance of data sharing and collaboration and key strategies for their implementation:

- Promoting open data sharing: Embracing a culture of open data sharing facilitates transparency, reproducibility, and innovation in biomedical research [44]. Biobanks can promote open data sharing by adopting data-sharing policies, releasing datasets to public repositories, and adhering to data sharing mandates from funding agencies or regulatory bodies. Open data sharing fosters collaboration, accelerates scientific progress, and increases the impact of research findings by enabling broader access to biobank resources.
- Establishing data access policies: Developing clear and transparent data access policies helps regulate access to biobank data while balancing privacy concerns, data governance requirements, and research needs [45]. Data access policies outline procedures for requesting, accessing, and sharing data, specifying eligibility criteria, data use restrictions, and compliance requirements. Implementing access control mechanisms, such as data use agreements and data access committees, ensures that data are accessed and used responsibly and ethically.
- Creating collaborative platforms: Establishing collaborative platforms and data-sharing portals facilitates communication, collaboration, and data exchange among researchers, biobanks, and other stakeholders. Collaborative platforms provide centralized access to data, tools, and resources, enabling researchers to discover, access, and analyze biobank data efficiently [46]. These platforms may include data repositories, virtual research environments, or collaborative networks tailored to specific research domains or disease areas.
- Data harmonization and integration: Harmonizing and integrating heterogeneous datasets from multiple biobanks or research studies enhances data interoperability and facilitates cross-study comparisons and meta-analyses. Collaborative efforts to standardize data formats, metadata annotations, and ontologies streamline data integration processes and enable researchers to aggregate, analyze, and interpret data from diverse sources effectively. Data harmonization initiatives promote data reuse, reduce redundancy, and maximize the value of biobank resources for research [3].
- Facilitating data-sharing agreements: Negotiating data-sharing agreements and collaborations with external partners, including academic institutions, industry partners, and international consortia, expands research opportunities and promotes knowledge exchange [47]. Data-sharing agreements delineate the terms and conditions governing data sharing, including data ownership, intellectual property rights, and data use restrictions, ensuring that data are shared responsibly and in compliance with legal and ethical requirements [48].
- Enabling federated data analysis: Federated data analysis approaches enable collaborative analysis of distributed datasets across multiple biobanks or research sites while preserving data privacy and security. Federated analysis platforms facilitate data aggregation, analysis, and knowledge discovery without centrally pooling or sharing sensitive data. By leveraging federated analysis techniques, researchers can collaborate on large-scale data analyses, identify patterns, and derive insights from diverse datasets while protecting participant privacy and data confidentiality.
- Promoting data citation and attribution: Encouraging data citation and attribution practices acknowledges the contributions of data contributors, promotes data reuse, and enhances research reproducibility and transparency. Providing persistent identifiers (DOIs) for datasets, citing data sources in publications, and adhering to data citation standards facilitate the proper attribution and recognition of data contributors. Data citation policies and guidelines promote responsible data use and incentivize data sharing within the research community.

## 6. Literature Reviews

This section traces the intellectual evolution of the data management field, highlighting significant debates and key references, with a particular emphasis on the techniques employed for general data management and their specific applications within biobanks. Furthermore, this review critically examines the limitations of each study, providing a more comprehensive analysis. It also evaluates the sources, identifying the most relevant and pertinent contributions to the field.

The reviewed paper [49] examines critical aspects of data management in biobanking, emphasizing the need for strong data privacy and security protocols to protect patient information, thereby maintaining public trust and meeting regulatory standards. The authors stress the importance of standardized data collection to enable cross-biobank comparisons, which are crucial for research and data sharing. Technological advancements in data storage, retrieval, and analysis are highlighted as essential for managing large datasets linked to biological samples. Effective data management is seen as key to advancing research and clinical applications, particularly in identifying biomarkers for personalized treatment in chronic disease care. The paper suggests future improvements should focus on enhancing data management policies and regulations. However, it also notes limitations, such as broad generalizations of challenges, insufficient discussions of specific technological solutions, and a lack of focus on ethical issues like informed consent and genetic data misuse.

The paper [50] introduces a data management system developed for the Andalusian Public Health System Biobank (SSPA Biobank) aimed at supporting personalized medicine. The model emphasizes data traceability and monitoring to improve research quality. However, the paper notes several challenges, including difficulties in integrating diverse data sources, maintaining compliance with international standards, and managing scalability as the biobank grows. The paper also highlights barriers to user adoption and training, as well as ongoing concerns about data privacy. Despite these challenges, the proposed model is robust, although addressing these limitations is crucial for its effective implementation and long-term sustainability.

The paper [51] emphasizes the critical role of effective data management in biobanks, particularly in oncology and translational medicine. It discusses how the proper organization and accessibility of biological samples and their data are vital for research. However, the variability in biobanking practices presents significant challenges to standardizing data management, including issues with data quality, consistency, and interoperability. These challenges can impede research and limit the use of biobank data across multiple studies. The authors propose that technological advancements and improved data management systems could address these issues, enhancing biobank efficiency and facilitating better data integration and sharing, especially in collaborative oncology research. Despite these solutions, the paper acknowledges ongoing limitations, such as inconsistent data quality, difficulties in achieving interoperability, and regulatory and ethical challenges in data sharing. Continued advancements focusing on standardization and collaboration are deemed essential for advancing translational oncology research and personalized medicine.

The paper [52] examines the ethical, legal, and societal (ELSI) challenges in biobank research, as reported by European professionals. The study highlights the need for improved informed consent processes, enhanced participant engagement, and stronger industry collaboration. However, the paper is limited by its focus on professionals’ perspectives, potential biases in the survey, a narrow scope of ELSI issues, and a lack of practical solutions for data management. It also does not fully consider the rapidly evolving landscape of biobank research. These gaps indicate that while the paper provides valuable insights, further research is necessary to address the broader complexities of data management in biobanking.

The paper [53] highlights the critical role of data management in healthcare, particularly in advancing medical data processing. It emphasizes the importance of analyzing large healthcare datasets using machine learning to uncover disease patterns essential for personalized treatment and prediction. The authors critique traditional medical storage systems, advocating for new models better suited to managing healthcare data. They stress the significance of effective noise reduction in medical imaging for accurate diagnosis and the role of AI in predicting diseases, noting that efficient data management is crucial for reliable predictive models. The paper reviews performance metrics for evaluating data management tools, underscoring their importance in accurate medical predictions. However, it identifies limitations such as inadequate storage systems, insufficient noise removal techniques, and the complexity of multi-disease prediction models. The authors call for improved frameworks to enhance data interoperability and collaboration, which are essential for advancing medical therapies and personalized medicine.

In the paper [54], the authors explore how effective data management can support personalized medicine, proposing an asymmetric encryption scheme with pseudonymization to protect patient privacy while linking clinical data with biomaterial samples. Although the approach is innovative, the paper points out several limitations, such as the complexity of implementing the system, potential conflicts among stakeholders with differing data access requirements, and the restricted scope of usable data. Additionally, concerns are raised about the technology’s ability to ensure data integrity and privacy, and the paper does not fully address the challenges of regulatory compliance. These limitations may impact the practical application and feasibility of the proposed data management strategies.

The paper [55] explores the advancements in biobanking, including new storage technologies and the introduction of diverse sample types. It emphasizes the need for updated processing methods and increased international collaboration. However, the paper falls short in providing detailed guidelines for implementing new data management practices, does not sufficiently address the integration of diverse data types, and offers only a limited discussion on ethical considerations. Additionally, it lacks a thorough outline of the practical implementation of new technologies and a clear research roadmap, which may hinder biobanks’ ability to adapt to future data management challenges.

The paper [56] emphasizes the critical importance of high-quality biological materials and data in medical research, particularly highlighting the need for well-documented and reproducible outcomes. While the paper discusses advances in maintaining the quality of biological samples through standardized procedures and thorough documentation, it points out that similar standards are often lacking in the management of data and metadata quality within biobanks. The authors outline the characteristics and requirements of effective data and metadata management systems, emphasizing that biobanks, which serve as both data producers and repositories, must implement robust quality assurance processes due to the sensitive nature of personal health data. However, the paper’s recommendations are mainly theoretical and are not backed by empirical evidence or case studies. Additionally, it does not thoroughly address practical challenges such as resource constraints, varying regulatory environments, or the impact of evolving data privacy regulations on managing personal health data within biobanks.

In the paper [57], the authors discuss the critical role of metadata in supporting medical research, particularly within biobanks. Metadata, defined as data that provide information about other data, are essential for ensuring the quality of both biological materials and the associated data. The paper identifies key quality attributes of metadata, including accuracy, consistency, coverage, timeliness, completeness, provenance, reliability, conformance, and accessibility. The authors propose metrics to assess these attributes as part of establishing effective metadata quality management systems. However, the paper has several limitations: it does not fully explore the definitions of metadata quality attributes, lacks a discussion on the volatility of metadata, and provides limited empirical validation for the proposed metrics. Furthermore, the paper postpones a discussion on ontology quality and raises concerns about the generalizability of the proposed characteristics across different biobanks. The authors call for further research to deepen the understanding of metadata quality and enhance its application within biobanks.

The paper [58] discusses the development of a harmonization toolkit designed to integrate colorectal cancer data from various European biobanks. The primary goal was to standardize data integration to enhance research quality by using a lexical bag-of-words matcher to align local biobank terminologies with a central standard. The tool successfully matched 78.48% of the data, processing information from over 3000 patients. However, the approach had limitations, such as the need for manual term mappings for unmatched items, data quality issues like inconsistent entries, and a narrow focus on colorectal cancer, which might limit its applicability to other types of biobanks. Although the tool is open-source and adaptable for further research, its effectiveness across different biobank structures remains uncertain.

In [59], the authors examine the application of statistical methods, such as statistical process control (SPC) and acceptance sampling plans (ASPs), to improve the accuracy of clinical databases. The paper uses case studies to illustrate these techniques and offers guidelines for selecting appropriate tools based on specific data and database characteristics. However, the study faces limitations, including the underutilization of statistical methods in clinical settings, a lack of managerial support, and communication gaps between data providers and users. Additionally, the documentation tailored for clinical contexts is limited, and the findings may not be generalizable due to small sample sizes. The guidelines provided may also require continuous updates, which could complicate their implementation.

The study [60] examines the difficulties in achieving a balance between data transparency and participant privacy. It highlights the necessity of anonymizing datasets to protect individual identities, noting that conventional anonymization methods may be insufficient, particularly with sensitive information. The paper presents the k-anonymity framework for evaluating re-identification risks, although it also recognizes the limitations of this approach, especially in the context of complex social science data. To bolster privacy, the authors suggest practical tools such as MinBlur and MinBlurLite, although their effectiveness across various scenarios remains to be fully confirmed. The paper stresses the importance of ongoing research to address evolving privacy issues and acknowledges that the proposed algorithms do not completely eliminate re-identification risks. Additionally, concerns are raised about the generalizability of the findings across different research areas and the subjective nature of classifying data sensitivity.

In [61], the authors explore strategies to improve data sharing in the field of neuroscience while maintaining participant privacy. The paper outlines several obstacles, such as persistent data privacy concerns, the uneven application of FAIR principles, and the challenges of international collaboration. It also addresses limitations in the technical infrastructure and the necessity for legal frameworks that support secure data access. The authors discuss the difficulties in preserving data consistency and retention in longitudinal studies, as well as the high resource demands of data management. Furthermore, the paper raises issues related to potential data misuse and the generalizability of findings across varied populations, emphasizing the need for adaptive data-sharing strategies as the field progresses.

The paper [62] introduces a model (BPDS) that leverages blockchain technology alongside privacy-enhancing methods to secure data sharing. This model combines blockchain’s capabilities for data traceability and immutability with federated learning and differential privacy techniques to provide strong privacy protection. Efficiency is boosted by employing gradient pruning to minimize communication overhead. While the model shows promise and scalability with real datasets, its performance in larger networks and diverse real-world situations has not yet been fully tested. The implementation complexity and the need to comply with global data protection regulations pose additional challenges. Despite its potential, the model requires further validation and practical application.

Ref. [63] examines the difficulties associated with sharing fragmented health data in the context of personalized medicine, emphasizing the role of biobanks in data integration and harmonization. It stresses the need to adhere to GDPR and FAIR principles to protect sensitive health information while facilitating effective data sharing. However, the paper also points out the challenges of harmonizing diverse datasets, maintaining privacy, and the constraints of existing frameworks like GDPR, which may limit flexibility. Issues such as data quality, the resource-intensive nature of data preparation, and the risk of information loss during aggregation are also discussed. These challenges highlight the need for better strategies and resources to enhance data sharing in personalized medicine.

Ref. [64] explores how to navigate the tension between sharing qualitative data and protecting participant privacy. The paper advocates for involving participants in the data-sharing process to better respect their privacy and self-image. It argues that de-identification and obtaining clear consent offer more effective privacy protection than anonymization, particularly in qualitative research involving detailed data. Although the paper provides valuable insights into participant trust and concerns about data reuse, its focus on a specific PhD project may limit the generalizability of its conclusions. It also highlights the challenges of achieving true anonymization in a digital age and suggests the need for further research, although it does not offer concrete policy recommendations or a comprehensive exploration of ethical frameworks.

The paper [65] presents a framework designed to navigate the complex challenges related to biobanking, particularly in collaborative and international research settings. It emphasizes crucial elements such as informed consent, the management of incidental findings, and the use of Transfer Agreements to ensure ethical and legal compliance. The authors propose a four-step checklist covering the study design, participant recruitment, sample handling, and results communication to promote adherence to ethical and legal standards. However, the checklist is primarily based on the H2020 B3Africa project, which may limit its relevance outside the EU. Additionally, it may not fully account for the varied legal and ethical frameworks globally, potentially restricting its applicability across different jurisdictions. The paper also focuses on compliance, which might overshadow the need for ongoing ethical reflection as biobanking practices evolve.

The report [66] examines the importance of maintaining high data quality within biobanks, particularly through accurate metadata, which supports effective research. It defines data quality in terms of reliability, usefulness, and accuracy, stressing the need for thorough documentation to ensure data provenance. The report advocates for implementing a robust quality management system to continually assess and document data quality, highlighting challenges such as health data sensitivity and privacy concerns. While the report provides a broad overview, it lacks empirical data and practical examples to substantiate its recommendations. Additionally, it does not thoroughly explore how privacy issues intersect with data quality management, leaving a gap in understanding how to balance these concerns in biobanking practices.

The paper [67] explores solutions for handling missing data in the Trauma Audit and Research Network (TARN) database, which includes 165,559 trauma cases. The authors report that 13.19% of cases have unknown outcomes and propose the use of non-stationary Markov models to address this issue, resulting in a revised mortality rate of 6.78%, compared to the naive estimates of 7.20% and 6.36%. The study highlights variations in mortality rates over time and across severity levels. While the proposed approach shows promise, its applicability may be limited to the TARN dataset, and the reliance on Markov models might not fully capture the complexities of patient outcomes. The paper also notes that alternative methods, such as multiple imputation, could introduce biases, indicating the need for further research to validate these methods in various healthcare settings.

The paper [68] reviews methods for addressing missing data in clinical studies and their implications for research validity. It classifies missing data into three categories: missing completely at random (MCAR), missing at random (MAR), and missing not at random (MNAR), each requiring distinct handling approaches. The paper examines various statistical techniques, including imputation, maximum likelihood estimation, and sensitivity analysis, and emphasizes the need for transparent reporting on how missing data are handled. However, the paper primarily provides theoretical insights with limited empirical examples and may not fully address the unique aspects of different studies. It also places a strong emphasis on statistical methods while overlooking the role of study design in minimizing missing data from the outset.

The paper [69] highlights the significance of maintaining high data quality in data warehousing for effective decision making. It underscores that poor data quality can lead to inaccurate analyses and suboptimal business decisions. The paper explores various mechanisms essential for identifying, correcting, and preventing data quality issues throughout the data lifecycle. While it offers a solid theoretical foundation for understanding data quality, it lacks extensive empirical evidence and case studies. Additionally, the paper does not address potential challenges such as resource limitations or industry-specific data governance standards. It also overlooks advancements in technology that could enhance data quality mechanisms, suggesting a need for further research into practical applications and emerging technologies.

The paper [70] explores the ethical and governance challenges related to biobanking within the framework of the Anti-Doping Administration Management System (ADAMS) overseen by the World Anti-Doping Agency (WADA). It identifies four key ethical concerns: the consent process, benefit sharing, the alignment of ethics and governance, and the use of doping control data for secondary research. The authors critique the current consent procedures, arguing that athletes may feel coerced into consenting to compete, creating ethical uncertainties. They propose a model that incorporates broad consent alongside iterative governance and stakeholder engagement to enhance ethical practices. The paper also critiques the WADA’s approach to harmonization, suggesting it lacks clarity and may impede effective global ethical governance. Recommendations include refining consent processes and ensuring accountability in secondary data usage to better balance athlete rights and research needs. However, the focus on anti-doping may limit the relevance of these models to other biobanking contexts, and empirical validation of the proposed models is necessary to assess their real-world effectiveness.

The paper [71] examines biobanking regulations in low- and middle-income countries (LMICs), highlighting their crucial role in biomedical research. It discusses challenges related to effective sample and data sharing, particularly emphasizing the need for informed consent in the context of higher disease burdens in LMICs. The BCNet initiative, spearheaded by the International Agency for Research on Cancer (IARC), aims to strengthen the biobanking infrastructure and provide educational support in these regions. The paper compares various laws and guidelines, revealing both ethical and legal challenges, and provides examples of effective governance systems. Nonetheless, its focus on BCNet countries may not fully capture global biobanking regulations, and it lacks empirical data and consideration of diverse cultural and socio-economic factors. Additionally, it does not address the role of private entities or the sustainability of biobanking amidst shifting political and economic landscapes.

Chapter [72] of book [73] provides an in-depth examination of data integrity and governance, emphasizing their role in ensuring data accuracy and compliance with regulations such as GDPR. Data integrity encompasses the accuracy, completeness, and consistency of data, while data governance involves managing data availability, usability, and security through internal policies and standards. The paper outlines essential processes, rules, and standards for maintaining data integrity and ensuring data protection against misuse. It also covers various applications across different sectors, demonstrating the importance of robust data governance in today’s data-centric environment. However, the paper lacks empirical evidence and case studies to support its theoretical frameworks and does not address the impact of emerging technologies or offer a comprehensive discussion on global regulatory frameworks. Its focus on GDPR compliance may limit its relevance to other data governance regulations.

The paper [74] investigates Swiss residents’ preferences regarding participation in personalized health research and data management. Conducted in September 2019 with a 34.1% response rate, the survey reveals that 39% of respondents preferred being contacted and reconsented for each new research project, while 52% favored anonymous data storage. The participants expressed a desire to retain ownership of their data and placed their trust in doctors and researchers to safeguard it. These findings suggest that aligning biobank and research institution governance strategies with public preferences could enhance participant willingness to share data. Although the study provides valuable insights into public attitudes, its findings may not be generalizable beyond the Swiss context.

The paper [75] outlines a framework designed to manage sensitive health data in compliance with ethical standards, particularly those specified by GDPR. The proposed framework emphasizes the establishment of a “Participation Pact” to foster trust between researchers and participants. It incorporates the roles of a Data Governance Board (DGB) and a Research Ethics Committee (REC) to ensure adherence to ethical and legal practices. Nonetheless, the framework’s focus on the Italian setting may limit its relevance to different regulatory contexts. Additionally, practical implementation challenges, such as resource limitations and variations in institutional support, may affect its effectiveness. The lack of extensive empirical evidence also means that the framework’s recommendations require further validation.

## 7. Future Directions

Looking ahead, the field of data management in biobanking is prepared for significant advancements and innovations. Here are some potential future directions:

### 7.1. Integration of Advanced Technologies

The integration of advanced technologies represents a promising future direction for biobanking, offering innovative solutions to enhance data management, analysis, and utilization in biomedical research [76]. Here, we explore the potential impact of advanced technologies on biobanking and the opportunities they present for driving scientific discovery and clinical translation [77,78]:

- Blockchain technology: Blockchain technology provides a decentralized and tamper-resistant platform for secure and transparent data management in biobanking [79]. By utilizing blockchain’s unalterable ledger and cryptographic hashing, biobanks can ensure data integrity, traceability, and auditability throughout the data lifecycle. Blockchain-based solutions enable secure data sharing, provenance tracking, and consent management, fostering trust among data contributors, researchers, and participants [80].
- Post-quantum cryptography and quantum-secure communication: To enhance data security against emerging threats posed by quantum computing, the integration of post-quantum cryptography (PQC) and quantum-secure communication technologies offers a promising path forward. These approaches are designed to counteract vulnerabilities that quantum computing could exploit, potentially compromising existing cryptographic systems.
  ○ Post-quantum cryptography: This involves developing cryptographic algorithms that are designed to stay secure even when quantum computers are in use. Unlike classical computers that use binary bits, quantum computers utilize qubits, which can exist in multiple states at the same time due to the principle of quantum superposition, allowing for significantly faster computations. This capability poses a threat to cryptographic methods such as RSA and Elliptic Curve Cryptography (ECC), which depend on the difficulty of solving mathematical problems like factoring large numbers or calculating discrete logarithms; these are tasks that quantum algorithms can handle much more efficiently. In biobanking, adopting PQC is vital to protect the vast amounts of sensitive personal and genetic data stored in these repositories. Given the potential for cyberattacks targeting personal identifiers and genetic sequences, PQC algorithms—such as those based on lattice-based cryptography, hash-based signatures, and multivariate quadratic equations—are being developed and standardized. Implementing these algorithms will help ensure that sensitive information remains secure, even as quantum computing becomes more widespread [81].
  ○ Quantum-secure communication: Quantum-secure communication uses the principles of quantum mechanics to safeguard data transmissions. Key techniques encompass Quantum Key Distribution (QKD) and quantum entanglement. QKD enables two parties to create a shared secret key protected by quantum laws. Any eavesdropping attempts would disturb the quantum states, making the intrusion detectable. For biobanks, using quantum-secure communication methods can greatly improve the protection of sensitive data during transmission. Given the frequent exchange of personal and genetic information among researchers, institutions, and regulatory bodies, ensuring the security and confidentiality of these communications is crucial. Technologies like QKD provide strong defenses against interception and tampering, thereby enhancing the security of data exchanges across networks [82,83].
- Artificial intelligence and machine learning: Artificial intelligence and machine learning algorithms enable biobanks to analyze large-scale datasets [84,85], identify patterns, and extract actionable insights for precision medicine and personalized healthcare [86]. AI-driven approaches facilitate data mining, predictive modeling, and biomarker discovery, accelerating the translation of biomedical research into clinical applications [87]. AI-powered decision support systems aid in clinical diagnosis, treatment optimization, and patient stratification based on genetic and clinical data [88,89].
- Federated learning: Federated learning facilitates collaborative model training across dispersed data sources while upholding data privacy and confidentiality. In biobanking, federated learning facilitates multi-center data analysis, enabling researchers to aggregate and analyze data from disparate biobanks without centrally pooling sensitive data. Federated learning platforms empower biobanks to collaborate on large-scale data analyses, share insights, and derive collective knowledge while protecting participant privacy and data security.
- Genomic data analysis: Advances in genomic technologies, such as next-generation sequencing (NGS) and single-cell sequencing, revolutionize genomic data analysis in biobanking [90]. High-throughput sequencing platforms generate vast amounts of genomic data, enabling the comprehensive characterization of genetic variation, gene expression, and epigenetic modifications. Bioinformatics tools and cloud-based analysis platforms facilitate genomic data analysis [13,91], variant interpretation, and genotype–phenotype association studies, advancing our understanding of complex diseases and guiding personalized medicine approaches [33].
- Omics integration: Integrating multi-omics data, including genomics, transcriptomics, proteomics, and metabolomics, offers holistic insights into biological systems and disease mechanisms [92]. Integrative omics analysis enables researchers to elucidate molecular pathways, identify biomarkers, and uncover therapeutic targets for precision medicine interventions [48]. Integrative bioinformatics approaches, such as pathway analysis, network modeling, and data fusion techniques, enhance data interpretation and facilitate discovery-driven research in biobanking [93].
- Biobanking informatics platforms: Biobanking informatics platforms provide integrated solutions for data management, analysis, and collaboration, streamlining biobank operations and supporting research workflows [45,94,95]. These platforms offer features such as sample tracking, metadata management, data curation, and analysis tools tailored to biobanking needs [26,96,97]. Cloud-based informatics platforms enable scalable and secure data storage, analysis, and sharing, empowering biobanks to leverage advanced technologies and collaborate with researchers worldwide [98].
- Emerging technologies: Emerging technologies, such as single-cell analysis, spatial transcriptomics, and organoid modeling, offer novel approaches for studying cellular heterogeneity, tissue architecture, and disease mechanisms in biobanking. These technologies enable researchers to capture fine-grained molecular profiles, spatially resolve cellular interactions, and model complex biological processes in vitro. Integrating emerging technologies into biobanking workflows expands research capabilities, facilitates disease modeling, and accelerates drug discovery efforts [99].

### 7.2. Long-Term Data Sustainability

Ensuring long-term data sustainability is a critical future direction for biobanking, aimed at preserving the integrity, accessibility, and usability of data resources for future research endeavors [4]. Here, we explore the importance of long-term data sustainability and strategies for its implementation:

- Data stewardship and governance: Establishing robust data stewardship and governance frameworks is essential for ensuring the long-term sustainability of biobank data [100]. Data stewardship involves the responsible management, curation, and preservation of data assets [101], while governance encompasses policies, procedures, and oversight mechanisms to ensure compliance with legal, ethical, and regulatory requirements. Implementing clear roles, responsibilities, and accountability structures fosters a culture of data stewardship and ensures the continuity of data management practices over time.
- Data preservation and archiving: Preserving data integrity and accessibility over the long term requires establishing archival strategies and preservation methods tailored to the unique characteristics of biobank data. Archiving data in secure, redundant storage systems, such as digital repositories or cloud-based storage solutions, safeguards against data loss, hardware failures, or technological obsolescence. Implementing data backup, versioning, and migration strategies ensures data resilience and facilitates data recovery in the event of system failures or disasters.
- Metadata standardization and documentation: Standardizing metadata formats, documentation practices, and data descriptors enhances data discoverability, interoperability, and usability over time [34]. Documenting metadata attributes, data provenance, and data processing protocols ensures that data remain comprehensible and interpretable by future users. Metadata standards, such as the Minimum Information About a Biobank (MIABIS) or the FAIR (Findable, Accessible, Interoperable, and Reusable) principles [30,101], guide metadata documentation and promote data sustainability by enhancing data reuse and interoperability.
- Data quality assurance and maintenance: Maintaining data quality and reliability is essential for preserving the value and integrity of biobank data over time. Implementing data quality assurance measures, such as regular audits, validation checks, and data cleaning procedures, ensures that data remain accurate, consistent, and fit for purpose. Ongoing surveillance of data quality metrics and performance indicators allows biobanks to detect and rectify instances of data degradation or quality issues proactively, thereby sustaining data utility and trustworthiness.
- Data security and privacy protection: Safeguarding data security and protecting participant privacy are paramount considerations for ensuring the long-term sustainability of biobank data [102]. Deploying strong data security measures, encryption techniques, access controls, and privacy safeguards helps alleviate the potential for data breaches, unauthorized access, or the misuse of data. Adhering to data protection laws, ethical guidelines, and best practices for data anonymization and de-identification ensures that data remain ethically and legally compliant while supporting data sharing and research collaboration.
- Community engagement and collaboration: Engaging stakeholders, including researchers, participants, funding agencies, and regulatory bodies, fosters collaboration, promotes transparency, and ensures the continued relevance and sustainability of biobank data resources. Soliciting feedback, addressing community needs, and involving stakeholders in decision-making processes empower stakeholders to contribute to data governance, policy development, and resource allocation efforts [103,104]. Collaborative initiatives, such as data-sharing consortia, working groups, and community-driven projects, foster a sense of ownership and collective responsibility for sustaining biobank data resources [105].

### 7.3. Ethical and Social Implications

Exploring the ethical and social implications of biobanking is crucial for guiding future directions in this field, ensuring that practices align with ethical principles [106], respect participant rights, and address societal concerns. Here, we delve into the ethical and social implications of biobanking and strategies for addressing them:

- Informed consent and participant autonomy: Upholding the principles of informed consent and participant autonomy is paramount in biobanking to ensure that individuals have the right to make informed decisions about the use of their biological samples and data [107]. Future directions should focus on enhancing consent processes, providing clear and understandable information to participants, and offering opportunities for dynamic consent, allowing individuals to update their preferences over time [108,109].
- Privacy and data confidentiality: Protecting participant privacy and ensuring the confidentiality of sensitive data are ethical imperatives in biobanking [110]. As biobanks collect and store large volumes of personal health information and genetic data, future directions should prioritize robust data security measures, anonymization techniques, and encryption protocols to mitigate privacy risks and prevent unauthorized access or breaches.
- Equitable access and benefit sharing: Addressing issues of equity and justice in biobanking involves ensuring that the benefits derived from research are shared equitably among participants, communities, and stakeholders. Future directions should promote transparent and fair access to biobank resources, prioritize the inclusion of under-represented populations in research, and establish mechanisms for benefit sharing, such as community engagement initiatives, research partnerships, and capacity-building programs.
- Data governance and oversight: Implementing effective data governance mechanisms and oversight frameworks is essential for ensuring responsible and ethical conduct in biobanking. Future directions should focus on developing robust data governance policies, establishing independent oversight bodies, and fostering collaboration among stakeholders to promote accountability, transparency, and ethical decision making in data management and research practices.
- Cultural sensitivity and respect for diversity: Recognizing and respecting cultural differences, values, and beliefs is essential in biobanking to ensure that research practices are culturally sensitive and inclusive [108]. Future directions should prioritize culturally tailored approaches to consent processes, engage with diverse communities in research planning and implementation, and address cultural concerns and preferences regarding data sharing, storage, and use [111].
- Public engagement and trust building: Building public trust and fostering the meaningful engagement of stakeholders are critical for success and sustainability of biobanking initiatives. Future directions should emphasize transparency, communication, and dialogue with the public, raise awareness about the benefits and risks of biobanking, and solicit input from diverse perspectives to inform decision-making processes and research priorities.
- Ethical use of biobank resources: Ensuring that biobank resources are used ethically and responsibly requires adherence to ethical guidelines, professional standards, and regulatory requirements. Future directions should prioritize ethical considerations in research design, data analysis, and the dissemination of findings, promote responsible conduct of research, and establish mechanisms for ethical review and oversight to safeguard participant welfare and uphold research integrity.

## 8. Conclusions

In conclusion, the management of data in biobanking presents a multifaceted challenge that requires careful consideration of technical, ethical, and regulatory dimensions. This review has highlighted key aspects of data management in biobanking, including data heterogeneity, quality assurance, privacy and security, governance, and regulatory compliance, as well as strategies for effective data management such as standardization, metadata annotation, and the integration of advanced technologies.

Biobanks serve as invaluable repositories of biological specimens and data, holding immense potential for advancing biomedical research, personalized medicine, and public health initiatives. However, realizing this potential necessitates addressing a significant number of challenges, from ensuring data quality and integrity to protecting participant privacy and complying with regulatory requirements.

Moving forward, it is imperative for biobanks to prioritize ethical principles, transparency, and stakeholder engagement in data management practices. Embracing open data sharing, collaboration, and responsible stewardship of data resources can foster trust, promote innovation, and maximize the impact of biobank initiatives on scientific discovery and healthcare delivery.

By addressing the complex landscape of data management challenges in biobanking and embracing emerging technologies and best practices, biobanks can position themselves as vital contributors to biomedical research and catalysts for transformative advancements in healthcare. Ultimately, the effective management of data in biobanking is not only a technical endeavor but also an ethical imperative that requires a holistic and interdisciplinary approach to ensure the responsible and sustainable use of valuable biological resources for the benefit of society.

## Data Availability

No new data were created or analyzed in this study. Data sharing is not applicable to this article.
